# Silencing of the potato *StNAC103* gene enhances the accumulation of suberin polyester and associated wax in tuber skin

**DOI:** 10.1093/jxb/erw305

**Published:** 2016-08-12

**Authors:** Roger Verdaguer, Marçal Soler, Olga Serra, Aïda Garrote, Sandra Fernández, Dolors Company-Arumí, Enriqueta Anticó, Marisa Molinas, Mercè Figueras

**Affiliations:** 1 Laboratori del Suro, Biology Department, Campus Montilivi, Girona, Catalonia, Spain; 2 Laboratoire de Recherche en Sciences Végétales, Université Toulouse III/CNRS, BP, Auzeville, Castanet Tolosan, France; 3 Chemistry Department, Faculty of Sciences, University of Girona, Campus Montilivi, Girona, Catalonia, Spain

**Keywords:** Apoplastic barriers, NAC transcription factor, periderm, phellem, regulation, StNAC103, suberin, suberin-associated wax, tuber

## Abstract

Suberin and wax deposited in the cork (phellem) layer of the periderm form the lipophilic barrier that protects mature plant organs. Periderm lipids have been widely studied for their protective function with regards to dehydration and for how they respond to environmental stresses and wounding. However, despite advances in the biosynthetic pathways of suberin and associated wax, little is known about the regulation of their deposition. Here, we report on a potato NAC transcription factor gene, *StNAC103*, induced in the tuber phellem (skin). The *StNAC103* promoter is active in cells undergoing suberization such as in the basal layer of the phellem, but also in the root apical meristem. Gene silencing in potato periderm correlates with an increase in the suberin and wax load, and specifically in alkanes, ω-hydroxyacids, diacids, ferulic acid, and primary alcohols. Concomitantly, silenced lines also showed up-regulation of key genes related to the biosynthesis and transport of suberin and wax in the tuber periderm. Taken together, our results suggest that StNAC103 has a role in the tight regulation of the formation of apoplastic barriers and is, to the best of our knowledge, the first candidate gene to be identified as being involved in the repression of suberin and wax deposition.

## Teaser

The silencing of *StNAC103*, a phellem-induced gene, reveals its repressor role in suberin and wax deposition and provides evidence of fine control over the formation of the apoplastic barrier.

## Introduction

The phellem or cork is a physical barrier at the interface between (mature) secondary growth tissues and the environment. It prevents dehydration and facilitates resistance to pathogens and mechanical wounds. The phellem is located within the outer face of the periderm, the dermal structure that replaces the epidermis in secondary stems, secondary roots, tubers, and tissues healing from wounds. The phellem (cork) cells undergo suberization, a process in which suberin and wax are deposited onto the inner face of primary cell walls during a differentiation process that ends with cell death (Pollard *et al.*, 2008). Suberin, like cutin, is a polyester consisting of fatty acids and glycerol forming a matrix in which wax is bound. However, in contrast to cutin, suberin contains longer chain length (>20 carbons) fatty acids and derivatives, a greater proportion of dicarboxylic acid monomers, and a covalently linked polyaromatic moiety (Bernards, 2002). The suberin-associated wax mostly consists of linear, very long chain aliphatic compounds of up to C32, including alkanes, primary alcohols, fatty acids, and feruloyl esters of primary alcohols. It has recently been shown in Arabidopsis roots that suberin-associated wax mainly consists of primary alcohols, predominantly in the form of alkyl hydroxycinnamates (Delude *et al.*, 2016). The role of both cuticular and suberin-associated wax in a reduction of water loss has been clearly demonstrated (Schreiber *et al.*, 2005; Zhang *et al.*, 2005; Kosma *et al.*, 2009; Lee *et al.*, 2014; Zhu *et al.*, 2014). Moreover, it has been shown that an increased production of cuticular wax leads to improved drought tolerance (Zhang *et al.*, 2005; Kosma *et al.*, 2009; Lee *et al.*, 2014; Zhu *et al.*, 2014). Analyses of Arabidopsis mutants with altered suberin accumulation demonstrated that increased suberin content resulted in better tolerance to salt stress and wilting (Beisson *et al.*, 2007; Baxter *et al.*, 2009; Gou *et al.*, 2009). The study of Arabidopsis suberin and wax mutants has facilitated the characterization of many enzymes involved in their biosynthetic pathways (for reviews, see Beisson *et al.*, 2012; Yeats and Rose, 2013). However, potato tuber skin has played an important part in the study of the influence of suberin and wax chemical composition on periderm physiology as sufficient amounts of periderm can be easily obtained. The main advantage of the potato periderm model is that it makes it possible to quantify water permeability to study the contribution of down-regulation of a gene in the water barrier properties of the periderm (Schreiber *et al.*, 2005; Serra *et al.*, 2009*a*, b, 2010; Landgraf *et al.*, 2014). For example, in potato tuber phellem, the silencing of *CYP86A33* greatly decreased suberin C18:1 diacids and ω-hydroxyacids, altered suberin lamellation, and resulted in a 3.5-fold higher permeability to water (Serra *et al.*, 2009*b*). In contrast, the silencing of *FHT* (*fatty ω-hydroxyacid/fatty alcohol hydroxycinnamoyl transferase*) resulted in a lack of feruloyl esters of primary alcohols in both suberin and associated wax, yielding potato tubers that were 15 times more permeable to water, although the suberin ultrastructure remained unchanged (Serra *et al.*, 2010).

Land plants commonly encounter adverse conditions that result in temperature, water, light, or salt stress, and have evolved regulatory mechanisms to adapt lipid barriers to changing environmental conditions. Plants respond to water deficit treatment by increasing the deposition of both leaf cuticular wax and cutin monomers (Kosma *et al.*, 2009). Moreover, it has been reported that Arabidopsis plants respond to salt stress by increasing the amount of alkyl coumarates in root wax (Kosma *et al.*, 2012). For cuticle regulation, a complex regulatory network responsive to developmental and environmental cues and mediated by hormones, transcription factors, and epigenetic and post-transcriptional mechanisms has been suggested (Yeats and Rose, 2013). In this regulatory network, abscisic acid (ABA) is thought to stimulate cuticle deposition together with a suite of AP2/EREBP, MYB, MADS-box, and HD-Zip IV transcription factors (Samuels *et al.*, 2008; Yeats and Rose, 2013; Hen-Avivi *et al.*, 2014). Little is currently known about the regulatory network controlling suberin and the associated wax. Wounding and abiotic stresses activate suberin and wax synthesis through the induction of genes such as *KCS* (*3-ketoacyl-CoA synthase*: Franke *et al.*, 2009; Lee *et al.*, 2009), *FAR* (*fatty acid reductase*: Domergue *et al.*, 2010), and *FHT* (Boher *et al.*, 2013). In healing tissues, suberin is deposited in suberizing cells of the wound-closing layer and the wound periderm. ABA induces suberin biosynthetic genes in Arabidopsis (Lee *et al.*, 2009; Barberon *et al.*, 2016), potato (Lulai *et al.*, 2008; Boher *et al.*, 2013), and tomato (Leide *et al.*, 2012), triggers suberin accumulation in potato tuber wound-healing tissue (Lulai *et al.*, 2008), and gives rise to ectopic suberin deposition in root (Barberon *et al.*, 2016). It has been shown that the application of ethylene leads to the disappearance of pre-formed suberin lamellae as well as to the finding that the plant’s nutritional status is key to the regulation of suberization, revealing an unexpected flexibility in the regulation (Barberon *et al.*, 2016). However, the transcription factors that regulate suberization are relatively unknown. Until recently, information was limited to the expression profile of specific regulatory genes induced in suberized tissues: an Arabidopsis AP2/ERF transcription factor (Lasserre *et al.*, 2008) and the cork oak (*Quercus suber*) and apple (*Malus×domestica*) transcription factors WRKY, NAM, and R2R3-MYB classes (Soler *et al.*, 2007, 2008; Almeida *et al.*, 2013; Legay *et al.*, 2015). The first and only evidence of a regulatory effect on suberin is *AtMYB41*, which ectopically activates the biosynthesis and assembly of suberin concurrently with the induction of suberin and lignin genes in Arabidopsis (Kosma *et al.*, 2014).

NAC transcription factors belong to one of the largest plant-specific transcription factor families and are represented by 110 genes in potato (Singh *et al.*, 2013). Typically, NAC proteins have a well conserved N-terminal region, known as the NAC domain, and a diversified C-terminal region known as a TAR (transcription activation region) domain (Olsen *et al.*, 2005). The NAC domain is involved in DNA binding whereas the C-terminal region acts in transcription regulation and protein–protein interactions. NAC transcription factors are involved in a diverse array of functions including development, lateral root formation, auxin signaling, secondary cell wall biosynthesis, and stress regulation (Olsen *et al.*, 2005; Zhong *et al.*, 2010; Puranik *et al.*, 2012). In potato, a large proportion of *NAC* genes are sensitive to abiotic stress (Gong *et al.*, 2015). In this study, we investigate the potato NAC transcription factor gene, *StNAC103* (Singh *et al.*, 2013), in tuber periderm. *StNAC103* is the putative ortholog to a cork oak NAM transcription factor gene highlighted as a phellem regulation candidate based on its up-regulation in cork oak phellem (Soler *et al.*, 2007) and its co-expression throughout the growing season with well-known suberin genes (Soler *et al.*, 2008). More recently, the putative ortholog of *StNAC103* in apple (*Malus×domestica*) was also identified in the skin of apple fruits showing russeting, a skin defect due to the accumulation of suberin on the fruit exocarp (Legay *et al.*, 2015). *StNAC103* belongs to the potato NAC subgroup C, which contains genes related to the stress response (*StNAC017* and *StNAC030*: Singh *et al.*, 2013) and organ development (*ATNAC4* and *CUC3*: Hibara *et al.*, 2006; Vidal *et al.*, 2013). The Arabidopsis putative ortholog of *StNAC103* (*ANAC058*) confers hypersensitivity to ABA and it has been suggested that it may be a modulator of ABA-mediated germination potential (Coego *et al.*, 2014). Here, we show that *StNAC103* is up-regulated in cells undergoing suberization and induced by wounding and ABA treatment. The silencing of *StNAC103* in potato periderm correlates with an increase in suberin and wax compounds concomitant with the up-regulation of suberin and wax genes, hence suggesting a negative regulatory effect on the deposition of apoplastic lipids. Taken as a whole, this work suggests that the periderm apoplastic barrier is finely regulated and that StNAC103 is involved in this regulatory network.

## Materials and methods

### Plant material

Potato plants (*Solanum tuberosum* Group Tuberosum) cvs Desirée and Andigena were propagated as described previously by Serra *et al.* (2010). *Solanum tuberosum* Group Tuberosum cv. Desirée was used for silencing. For Desirée tubers, *in vitro* plants grown in MS (Murashige and Skoog) medium supplemented with 2% sucrose were transferred to soil and grown for 3 months in a walk-in chamber before harvesting. Andigena plants were used for detection of the promoter activity given that in this cultivar tuber onset is induced by photoperiod and tuber maturation can be more finely regulated than for Desirée plants. For Andigena plants, tuberization was induced *in vitro* by growing two-node stem explants in MS medium supplemented with 8% sucrose in short-day conditions (8h light/16h dark) at 22 ºC for 1 week and then transferred to the dark. We also used *S. tuberosum* Group Tuberosum cv. Monalisa potatoes, purchased at a local supermarket, for the wound healing experiment. For this experiment, potato tuber discs (3mm thick and 13mm in diameter) were obtained by cutting tuber flesh (parenchyma) excised with a cork borer and left to heal at room temperature in saturated humidity and dark conditions.

### ABA treatment of roots in hydroponic cultures

Desirée plants were propagated first *in vitro* and after 3 weeks plants were transferred to hydroponic culture and grown for 15 d in half-strength Hoagland**’**s solution (Arnon and Hoagland, 1939) in a 10 liter container in a growth chamber (light/dark photoperiod cycle of 12/12h at 22 ºC and 67 µmol m^−2^ s^−1^). Subsequently, a solution of ABA (Sigma, A-1049) suspended in ethanol was added to the cultures at a final concentration of 50 µM and roots were incubated for 5h and 26h. Control plants were treated with a mock solution of ethanol. Each time point had 4–6 biological replicates, each consisting of a pool of three roots from different plants.

### RNA extraction of potato tissues

Periderm tissue was manually dissected using sterile scalpels. Once harvested, tissue samples were immediately frozen in liquid nitrogen. Total RNA was isolated using the guanidine hydrochloride method (Logemann *et al.*, 1987) and genomic DNA was removed by on-column DNase digestion using the RNeasy MinElute (Qiagen) in accordance with the manufacturer’s instructions. RNA concentration and purity were determined by formamide–formaldehyde denaturing agarose gel electrophoresis and a Nanodrop spectrophotometer. First-strand cDNA was synthesized from 2 µg of DNase-digested RNA. We used the Superscript III reverse transcriptase (Invitrogen) with oligo(dT)_18_ primers for the synthesis of full-length cDNA before cloning, or the high capacity cDNA reverse transcription kit (Life Technologies) with random primers to synthesize cDNA for real-time reverse transcription–PCR (RT–PCR) analysis.

### Recombinant vector construction and plant transformation

Since information about the potato genome was not available at that time, primers to amplify and clone the *StNAC103* coding sequence and the region used for silencing were designed using the information available from the potato Expressed Sequence Tag assembly (TC143904) with homology to the cork oak sequence (EE743827) encoding a NAC transcription factor isolated from a SSH phellem library by Soler *et al.* (2007). We also used the most similar sequences from Arabidopsis (At3g18840) and *Petunia×hybrid* (GI:21389167) to design primers when potato sequence information was not available. For *StNAC103* silencing, a specific fragment of 253bp located primarily in the TAR domain was used. The amplification product was first cloned into the pENTR/D TOPO vector (Life Technologies) and then transferred into the binary destination vector pBIN19RNAi (Serra *et al.*, 2009*b*) using LR clonase II (Invitrogen). Using the potato genome sequence available, specific primers were designed to get a 2291 bp sequence upstream of the start codon (primers are shown in Supplementary Table S1 at *JXB* online). The PCR product was then inserted into the pDONR207 vector using the BP clonase II (Life Technologies) and subsequently transferred to the vector pKGWFS7 (Karimi *et al.*, 2002) using LR clonase II.

Recombinant plasmids were inserted into *Agrobacterium tumefaciens* (GV2260) following the protocol of Höfgen and Willmitzer (1988), and leaves were infected with transformed *Agrobacterium tumefaciens* cells. Kanamycin-resistant plants were regenerated *in vitro* by organogenesis according to Banerjee *et al.* (2006).

### Histochemical GUS staining of Pro*StNAC103*:*GUS-GFP* transgenic plants

β-Glucuronidase (GUS) histochemical staining was performed using Andigena transgenic potato plants containing the Pro*StNAC103*:*GUS-GFP* construct. Fixation was done by immersion of the transformed tissue in an ice-chilled 90% acetone (v/v) bath, followed by incubation for 20min on ice and rinsing twice with water before infiltration for 20min under vacuum with the staining solution [1mM 5-bromo-4-chloro-3-indolyl-β-d-glucuronic sodium salt 3·H_2_O (X-GlcA, Duchefa), 50mM sodium phosphate buffer (pH 7), 1.25mM potassium ferrocyanide, 1.25mM potassium ferricyanide, 10mM EDTA, and 0.05% (v/v) Triton X-100]. Incubation in staining solution was extended for a maximum of 48h at 37 ºC and then cleared with 70% (v/v) ethanol. Transformed GUS-stained and control tissues were examined with a bright-field microscope (AH2 Vanox-T microscope Olympus) and a stereomicroscope (Wild M420 Makroskop), and photographed with a digital camera (Olympus Camedic C-4040ZOOM).

### Real-time PCR analysis

Real-time PCRs were performed in an optical 96-well plate with an ABI PRISM 7300 Sequence Detector System (Applied Biosystems) and a Fluidigm (Biomark). Gene-speciﬁc PCR primers were designed with Primer 3 (Untergasser *et al.*, 2012) and checked with Primer Express 3.0 (Applied Biosystems). Reactions contained 1× Power SYBR Green Master Mix reagent (Applied Biosystems), 300nM of the respective primers, and 5 µl of a 25-fold dilution of the corresponding cDNA in a ﬁnal volume of 20 µl. The following standard thermal profile was used for all PCRs: 95 °C for 10min; 40 cycles of 95 °C for 15s and 60 °C for 1min. A dissociation step was performed after amplification to confirm the presence of a single amplicon. All data were processed and analyzed with 7300 SDS 1.3.1 software (Applied Biosystems). For microfluidics quantitative PCR (qPCR; Fluidigm), 1.25 μl of the synthesized cDNA was pre-amplified and then purified with exonuclease treatment. Pre-amplified and purified cDNAs were diluted to 1:5 and used for real-time qPCR amplification the BioMark™ system (Fluidigm). A dissociation curve was obtained to check primer specificity for each amplification reaction. Data collection and analysis was performed using Fluidigm Real-Time PCR analysis software 3.0.2 (Fluidigm). For each primer pair, standard curves with a 5-fold dilution series (1/1, 1/5, 1/25, 1/125, 1/625) of template were obtained to determine the amplification efficiency.

mRNA abundance was calculated as relative transcript abundance=(E_target_)^ΔCt target (control–sample)^/(E_reference_)^ΔCt reference (control–sample)^ (Pfaffl, 2001). The controls used to standardize data were as follows: for the *StNAC103* transcript accumulation pattern in native tissues, a mix with equal amounts of all samples; for transcript accumulation in tuber wound healing time course, a pool of each biological replicate was obtained at 144h post-wounding; for wound-healing leaves, a pool of each biological replicate was obtained at 72h post-wounding; for transcript accumulation in silenced transgenic lines, a pool of biological replicates of the control line (Desirée) was used. The housekeeping gene *APRT* encoding an adenine phosphoribosyl transferase was used to normalize the results (Nicot *et al.*, 2005; Soler *et al.*, 2011), except for the time course experiment of the tuber discs in which the constitutive gene *EF1α* was employed as it has less variation in these conditions. To check the absence of genomic DNA contamination, reverse transcriptase controls were used, whereas to check the absence of environmental contamination we used non-template controls (NTCs). The sequence of primers used for real-time PCR is shown in Supplementary Table S1.

### Isolation of periderm membranes

Periderm membranes used for chemical and water permeance analyses consist of the phellem (cork) layer after removal of the non-suberized tissue by enzymatic digestion as described by Schreiber *et al.* (2005). In short, fragments of tuber skin were separated from the inner flesh with a cork borer and incubated in a 2% (v/v) cellulase and a 2% (v/v) pectinase solution to remove all unsuberized (parenchymal) cells. After digestion, the remaining phellem membranes were washed, dried, and stored at room temperature until used. All membranes were obtained from tubers after 1 month storage at room temperature to allow post-harvest periderm maturation.

### Suberin and wax chemical analyses

Suberin and wax analyses were performed following the procedure described by Schreiber *et al.* (2005). Briefly, the wax fraction was extracted by a treatment with a mixture of chloroform and methanol (1:1 v/v) for 18h. For depolymerization of the aliphatic suberin, wax-free membranes were then trans-esterified by incubation at 70 ºC for 18h with methanol/boron triﬂuoride (~10% BF_3_ in methanol; Fluka). All compounds were quantiﬁed and analyzed as trimethylsilyl (TMS) derivatives, obtained by *N*,*O*-bis (trimethylsilyl) triﬂuoroacetamide (BSTFA; Macherey-Nagel) derivatization. Derivative products were identified by GC with a selective mass detector with ion trap (Trace GC 2 000 series coupled to a Thermo Scientific Polaris Q a mass spectrometer). For each peak, the mass spectrum was compared with data reported in the literature (Kolattukudy and Agrawal, 1974; Zeier and Schreiber, 1997; Zeier and Schreiber, 1998). Quantification was performed using a GC-FID (flame ionization detector; Shimadzu GC- 2010 Plus, Kyoto, Japan) by comparison of peak areas with an internal standard [for wax compounds, tetracosane (Fluka); for suberin monomers, dotrioacontane (Fluka)]. Results were statistically analyzed using Student’s *t*-test.

### Measurement of peridermal permeance

Peridermal permeance to water was measured using transpiration chambers by a gravimetric method (Schreiber *et al.*, 2005; Serra *et al.*, 2009*b*). Weight loss was measured at regular time periods during 30 d in an analytical balance. Water permeance (P, m s^−1^) was obtained using only the slope values from chambers in which water loss (g) across the periderm against time (s) fit a linear regression of *R*^2^ ≥0.99.

### Accession numbers

Accession numbers of the *StNAC103* promoter region (2291bp upstream of the translation initiation codon) and the *StNAC103* full-length coding sequence of the *S. tuberosum* Group Tuberosum are KT582103 and KT598221, respectively.

## Results

### *StNAC103* is expressed in suberized potato tissues

**Fig. 1.**
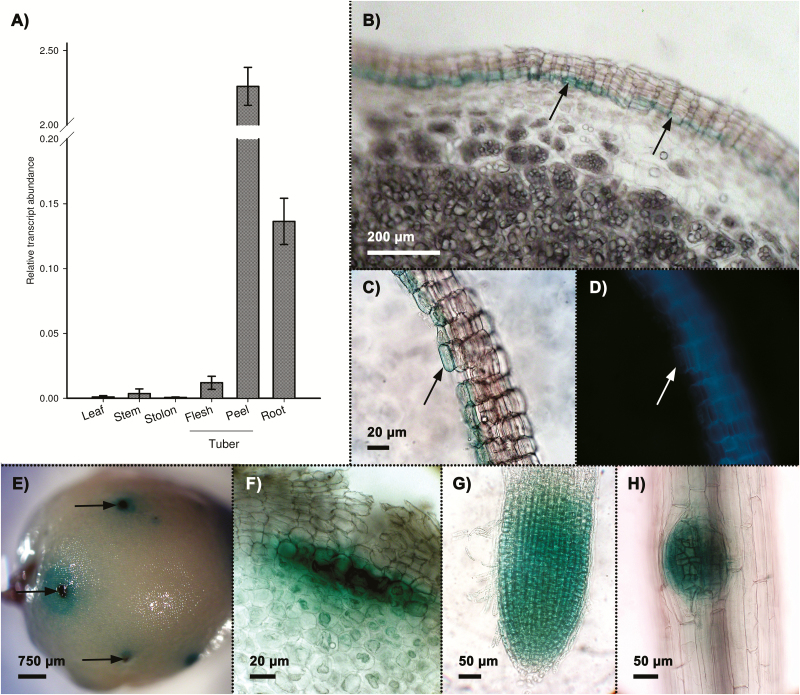
*StNAC103* transcript accumulation and promoter activity in potato native tissues. (A) Transcript abundance in different potato organs and tissues. Relative mRNA expression levels were expressed as the mean ±SD (the biological replicates were four for stem, leaf, and root, three for stolon and parenchyma, and two for phellem). (B–F) *StNAC103* promoter activity in Pro*StNAC103*:*GUS-GFP* tuber. (B) Section of a tuber showing GUS staining specific to the inner phellem cell layers (arrows). (C) Sheet of mechanically isolated phellem showing the GUS blue marker and the (D) suberin autofluorescence. (E) The GUS signal observed through the tuber surface showing lenticels as dark blue dots (arrows). (F) Detail of a lenticel seen in cross-section showing the GUS blue marker located to the lenticular phellogen. (G, H) Root whole mounts showing the GUS signal localized in (G) apical and (H) lateral root meristems.

The protein sequence of the *S. tuberosum* Group Tuberosum StNAC103 (KT598221) shares 98.41% identity with StNAC103 from *S. tuberosum* Group Phureja (Supplementary Fig. S1A). Both sequences are identical in the conserved NAC domain, with the differences restricted to the TAR domain. The expression profile of *StNAC103* Group Tuberosum in vegetative tissues was analyzed by quantitative RT–PCR; transcripts were mainly detected in tissues in which suberin is constitutively present, specifically tuber skin (phellem) and root (Fig. 1A). To analyze *StNAC103* expression further, potato plants were stably transformed with a construct containing the *StNAC103* promoter region fused to the coding sequence of the double transcriptional reporter GUS–green fluorescent protein (GFP). Tubers bearing the *ProStNAC103*:*GUS-GFP* were analyzed for GUS activity. The blue color, which is indicative of promoter activity, was localized in the periderm and restricted to the internal (basal) cell layers of the phellem (Fig. 1B). This localization was confirmed in skin sheets containing only the phellem peeled off from the tubers (Fig. 1C). Phellem cells in which the promoter was active corresponded to living cells undergoing differentiation but in which suberin autofluorescence is very faint or absent (Fig. 1D, arrow). GUS staining of whole tubers revealed large areas of the tuber surface with weak GUS expression, but also high GUS expression corresponding to the lenticels (Fig. 1E, arrows). The deep blue labeling of lenticels matched with the lenticular phellogen at the base of the lenticel (Fig. 1F). *StNAC103* promoter activity was also analyzed in primary roots obtained from hydroponic cultures in which promoter activity was not localized in the suberized root barriers, exodermis, or endodermis, but in lateral root primordia and root apical meristems (Fig. 1G, H). Such a pattern contrasts with that of *FHT*, a well-studied gene involved in suberin biosynthesis (Serra *et al.*, 2010) which is active only in suberized cell layers (Boher *et al.*, 2013). Both patterns can be compared in Supplementary Fig. S2, showing potato roots from plants bearing, respectively, the *ProStNAC103:GUS-GFP* and the *ProFHT:GUS-GFP* constructs, grown and stained in parallel.

### *StNAC103* expression is triggered by wounding stress and ABA treatment

**Fig. 2.**
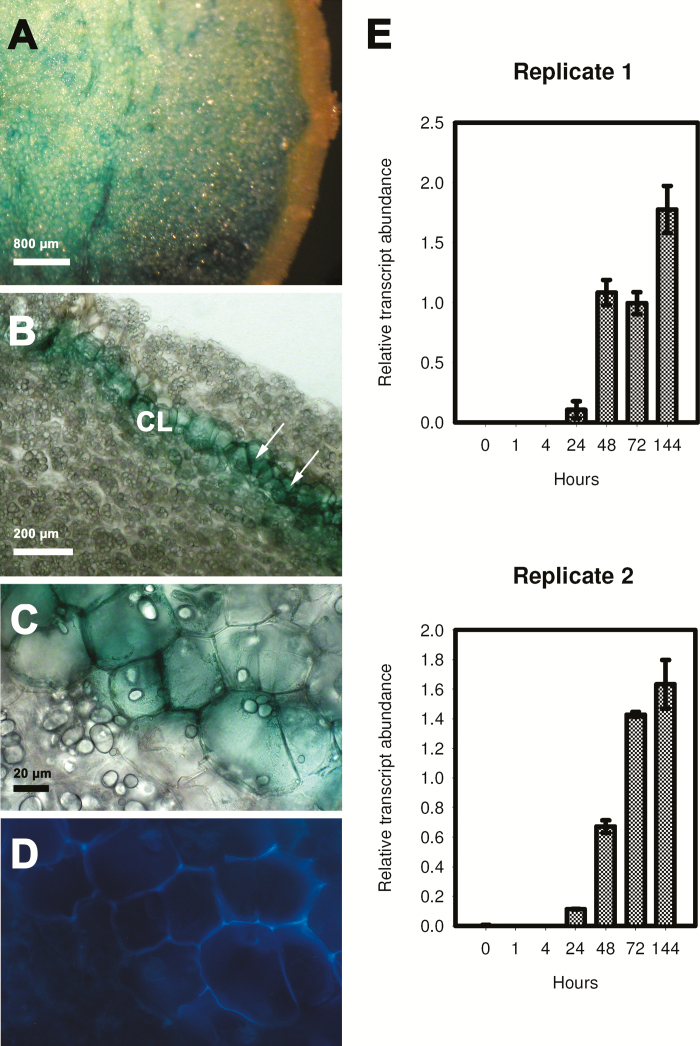
*StNAC103* expression in wound-healing tubers. (A–D) *StNAC103* promoter activity in Pro*StNAC103:GUS-GFP* plants. (A) GUS staining of wound-healing tubers 48h after wounding observed through the cut surface. (B) In a section through the cut surface, the blue color localized to the wound-healing tissue (arrows). (C) Detail of (B) under bright field and (D) under UV excitation to indicate suberin autofluorescence. (E) Time course accumulation of *StNAC103* transcript in potato tuber healing discs during 144h presented separately for two biological replicates. The mean ±SD of three technical replicates is shown. CL, closing layer.

**Fig. 3.**
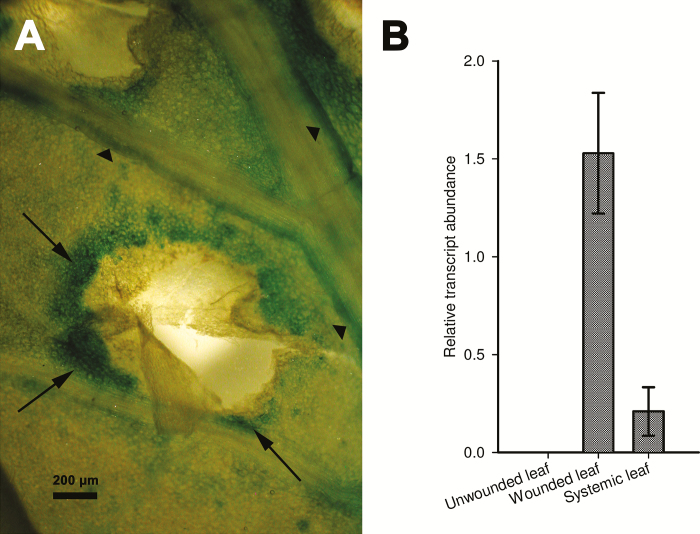
*StNAC103* in wound-healing leaves. (A) GUS staining of a leaf 72h after wounding showing the blue color in the wound margins (arrows) and in a nearby minor vein (arrowheads). (B) *StNAC103* transcript accumulation in wounded, unwounded, and systemic leaf is reported. Relative mRNA expression levels were expressed as the mean ±SD of two biological replicates.

**Fig. 4.**
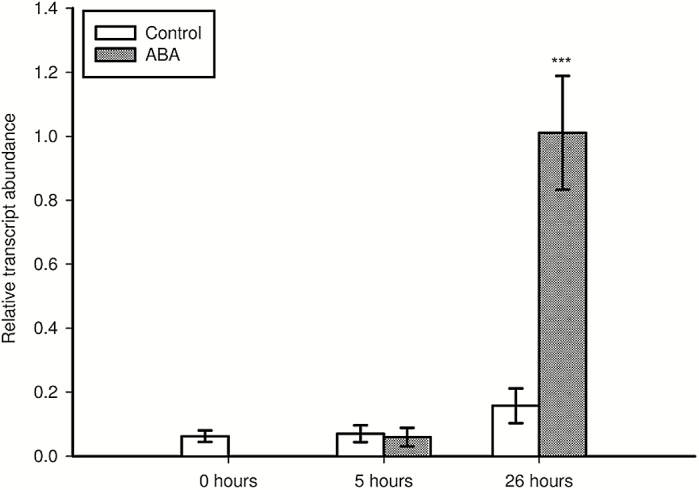
*StNAC103* induction by ABA in roots grown in hydroponic culture. The transcript abundance was analyzed in roots of plants treated with 50 µM ABA during 5h and 26h. Relative mRNA expression levels were expressed as the mean ±SD of 4–6 biological replicates. Pronounced statistically significant differences are shown with three asterisks (*P*<0.001). Results were statistically analyzed using Student’s *t*-test.

To examine the activation of *StNAC103* in healing tissues, we injured tubers and leaves in *ProStNAC103:GUS-GFP* plants. After 48h of wounding by a single cut, tubers showed reporter expression at the severed surface (Fig. 2A). Microscopic examination revealed that it was confined to the cell layers in which suberization occurs (Fig. 2B). In healing tissues, GUS-stained cells are living cells depleted of starch grains (Fig. 2C) whose cell walls display the typical suberin autofluorescence under UV excitation (Fig. 2D). A time course quantitative RT–PCR analysis of *StNAC103* expression throughout the healing process was performed in healing tuber flesh (parenchyma) discs obtained from commercial potatoes. *StNAC103* transcripts were detected beginning at 24h after injury, and levels increased gradually during the wound healing (Fig. 2E). Expanded leaves were also injured by pinching the leaflets with tweezers and then left to heal. In healing leaves (72h after wounding), the GUS activity was mainly restricted to the wound margins (Fig. 3A, arrows) but was also detected in some surrounding tissues (Fig. 3A, arrowheads). To evaluate a systemic activation of the promoter as a consequence of a local wounding, we compared *StNAC103* transcript levels in damaged leaves with those of undamaged leaves on the node immediately below (systemic leaves) and also with the expression in leaves of unwounded plants. Both damaged and systemic leaves showed transcript accumulation, although levels were lower in systemic leaves (Fig. 3B). We further investigated *StNAC103* induction in response to ABA in potato roots grown in hydroponic cultures. As can be seen in Fig. 4, after 26h of ABA treatment (50 µM) there was a strong accumulation of *StNAC103* transcript in roots. Hence, this gene is also inducible by abiotic stress signals such as wounding and ABA, and this responsiveness occurs in roots but also in tissues where *StNAC103* is not preferentially expressed, such as tuber flesh and leaf.

### *StNAC103* silencing

**Fig. 5.**
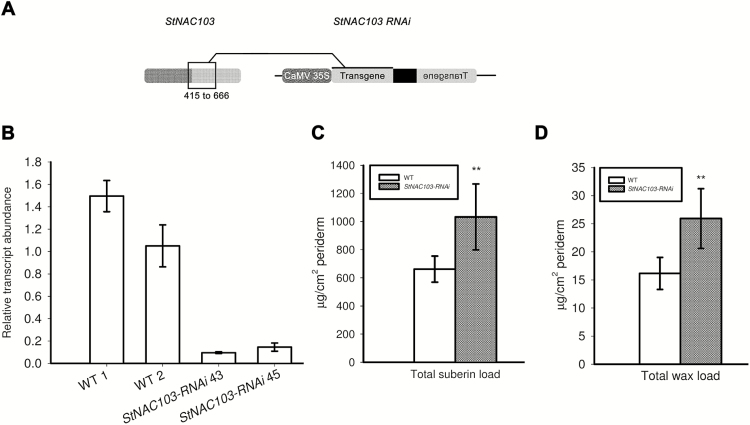
Effects of *StNAC103* silencing in the apoplastic lipid load of the tuber periderm. (A) Scheme of the *StNAC103-RNAi* construct designed for gene silencing targeting the variable TAR doamin. The NAC domain is shown in dark gray and the TAR domain in light gray. (B) *StNAC103* relative transcript abundance in the periderm of silencing and wild-type lines. Relative mRNA expression levels were expressed as the mean ±SD of three technical replicates. (C) Total suberin and (D) total wax load in the wild-type and *StNAC103-RNAi* periderm from tubers stored for 39 d. Significant differences are shown with two asterisks (*P*<0.01).

To evaluate the role of *StNAC103* in the phellem, we generated transgenic potato plants that silence *StNAC103*. The silencing construct was targeted primarily to the variable TAR domain, the most specific region of the *StNAC103* gene, and was designated as *StNAC103-RNAi* (Supplementary Fig. S1B; Fig. 5A). In order to check the specificity of the silencing, putative targets of the *StNAC103-RNAi* construct were investigated by a blastN performed in the potato genome database (Potato Genome Sequencing Consortium *et al.*, 2011; PGSC S. tuberosum group Phureja, DM1-3 Transcripts v3.4, expected threshold of 1) using the silencing RNAi sequence as a query. The blastN identified the *StNAC103* transcript with 0 mismatches along all the query sequence and a partial match with *StNAC057* in one region with two mismatches in 21 consecutive nucleotides. To check if despite these two mismatches *StNAC057* could be an off-target of the sequence used for RNAi silencing, the relative transcript abundance of *StNAC057* was analyzed in *StNAC103-RNAi* and wild-type periderms (Supplementary Fig. S3). No decrease was observed in *StNAC057* transcript levels in the *StNAC103-RNAi* periderms, so ruling out cross-silencing.

*StNAC103* gene silencing in tuber periderm was confirmed by RT–PCR in a number of kanamycin-resistant lines, and those lines showing a substantial decrease in transcript levels were selected (Fig. 5B). All potato plants deficient in StNAC103 developed normally either *in vitro* or in pots. Likewise, transgenic tubers displayed no visual differences compared with the wild type during tuber development, at harvest, or during storage. Two *StNAC103-RNAi* lines (43 and 45) corresponding to different transformation events were propagated for tuber production and used for phenotypic analyses.

### *StNAC103* impacts the suberin and wax composition of the potato tuber periderm

**Fig. 6.**
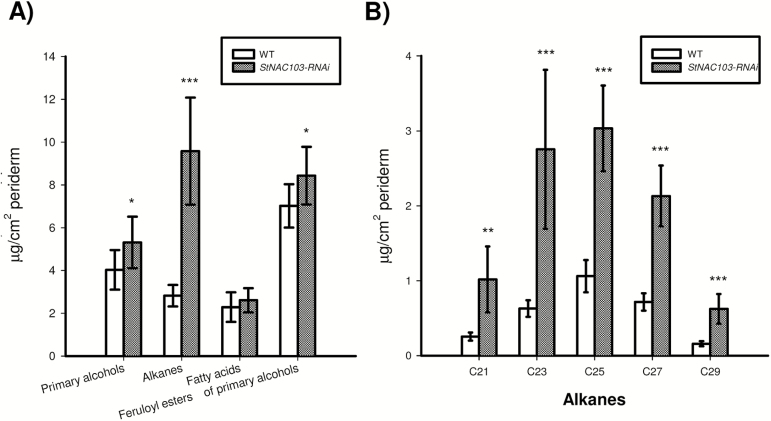
Effects of *StNAC103* silencing on the wax chemical composition shown as the amount of compounds per surface area from tuber periderm stored for 39 d. (A) Composition by substance classes of the wild-type and *StNAC103-RNAi* periderm. (B) Profile of alkanes identified in the periderm of wild-type and silencing lines. Values are the the mean ±SD of the wild type (five biological replicates) and two independent transformation events for *StNAC103-RNAi*, lines 43 and 45 (four and three biological replicates, respectively). Significant differences (*P*<0.05) are denoted with one asterisk, whereas pronounced significant differences (*P*<0.01; *P*<0.001) are shown with two and three asterisks, respectively.

**Fig. 7.**
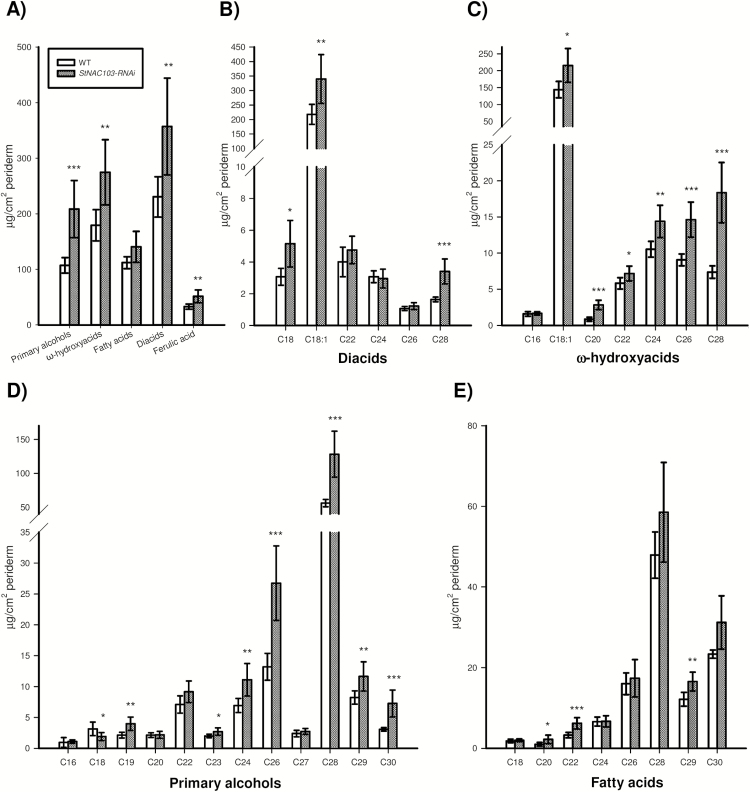
Effect of *StNAC103* silencing on suberin composition shown as the amount of compounds per surface area from tuber periderm stored for 39 d. (A) Composition by substance classes, (B) profile of diacids, (C) profile of ω-hydroxyacids, (D) profile of primary alcohols, and (E) profile of fatty acids represented by their carbon chain length of the wild type and *StNAC103-RNAi* lines. Values are the mean ±SD of the wild type (five biological replicates) and two independent transformation events for *StNAC103-RNAi*, lines 43 and 45 (four and three biological replicates, respectively). Significant differences (*P*<0.05) are denoted with one asterisk, whereas particularly pronounced significant differences (*P*<0.01; *P*<0.001) are shown with two and three asterisks, respectively.

The effect of *StNAC103* on the chemical composition of the suberin polymer and solvent-extractable wax was analyzed by GC-MS and quantified by GC-FID from enzymatically isolated periderm membranes obtained as described in the Materials and methods. Tubers deficient in StNAC103 showed a significant increase in wax and suberin load and important changes in their composition (Fig. 5C, D). The total amount of each wax substance class showed that the most affected substance class is that of alkanes (Fig. 6A), which significantly increased in the periderm of silenced lines. Primary alcohols and feruloyl esters of primary alcohols were also affected. The chemical composition analysis of the wax fraction showed that individual alkanes doubled their amounts in *StNAC103-RNAi* periderm, compared with the wild type (Fig. 6B), while feruloyl esters of primary alcohols and primary alcohols showed smaller differences between the wild type and silenced periderms (Supplementary Fig. S4). As can be seen in Supplementary Fig. S4, only C22 and C28 primary alcohols increased. Regarding feruloyl esters of primary alcohols: C16, C18, and C20 decreased and C23, C24, C26, and C30 increased. For the suberin fraction (Fig. 7A), *StNAC103* silencing resulted in an increase in the primary alcohols, ω-hydroxyacids, diacids, and ferulic acid. Amongst single monomers, the levels of the two main monomers in the suberin from potato periderm, C18:1 diacid and C18:1 ω-hydroxyacid, were increased (Fig. 7B, C), but the C18 and C28 diacid were also augmented. In addition, all the ω-hydroxyacids longer than C18 were also increased in the silenced lines (C20, C22, C24, C26, and C28 ω-hydroxyacids). Among primary alcohols, the amounts of C19, C23, C24, C26, C28, C29, and C30 alcohol were enhanced, but the C18 alcohol decreased (Fig. 7D). Results for the separate lines are plotted in Supplementary Figs S5 and S6. The significant role of StNAC103 in periderm lipids led us to investigate its contribution to the water barrier function. To this end, we analyzed the water permeance in enzymatically isolated periderm membranes using a gravimetric method. As shown in Supplementary Fig. S7, the permeance to water was not statistically different between *StNAC103-RNAi* and wild-type periderms, and thus the increase in suberin and wax load was not found to play a significant role in the physiology of the periderm.

### *StNAC103* silencing results in the induction of some genes key for suberin and wax accumulation

**Fig. 8.**
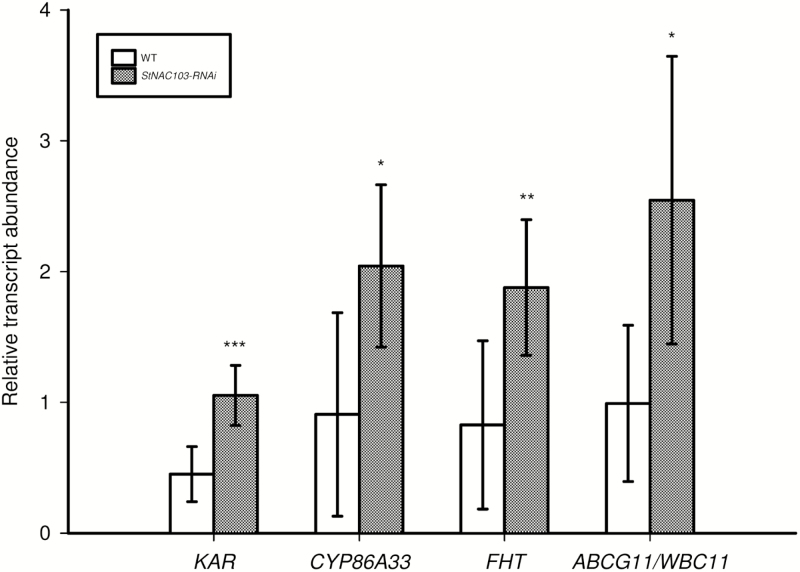
Microfluidics qPCR in *StNAC103-RNAi* and wild-type periderm of some key genes involved in suberin and wax accumulation. The relative transcript abundances of *KAR*, *CYP86A33*, *FHT*, and *ABCG11/WBC11* are shown. Values are the mean ±SD of the wild type (four biological replicates) and two independent transformation events for *StNAC103-RNAi*, lines 43 and 45 (five and four biological replicates, respectively). Significant differences (*P*<0.05) are denoted with one asterisk, whereas pronounced significant differences (*P*<0.01; *P*<0.001) are shown with two and three asterisks, respectively.

To understand further the molecular mechanisms of StNAC103 function, the relative expression of a selected set of genes was analyzed by microfluidics qPCR in silenced and wild-type periderms. The genes analyzed were representative of *de novo* synthesis of fatty acids (*KAR*, ketoacyl-ACP reductase), ω-hydroxylation of fatty acids (*CYP86A33*, cytochrome P450), cross-linking of the fatty acid polyester to the ferulic acid (*FHT*, fatty ω-hydroxyacid/fatty alcohol hydroxycinnamoyl), and export of suberin and wax compounds (*ABCG11/WBC11*). Relative transcript abundance shows that all these genes are up-regulated in the periderm of *StNAC103*-*RNAi* lines in comparison with the wild type (Fig. 8), and hence they act downstream of StNAC103. As such they are either direct or indirect targets of this transcription factor.

## Discussion

The *StNAC103*-silenced potato plants show an increase of the total load of suberin and wax in the periderm. In the wax fraction, the silencing mainly produces an increase in the alkane accumulation whereas in the suberin fraction there is an increase in the ω-hydroxyacids, diacids, primary alcohols, and ferulic acid. In agreement with this phenotype, some key genes that are representative of the biosynthesis and transport of suberin and wax display up-regulation in the periderm of *StNAC103-RNAi* lines. All of the above suggest a repressor role for StNAC103 in the suberin and suberin-associated wax. Together with *AtMYB41*, *StNAC103* is the second transcription factor characterized with a clear effect in apoplastic lipids (Kosma *et al.*, 2014). However, *AtMYB41* works as an activator showing a correlation between gene overexpression, up-regulation of suberin-related genes, and ectopic deposition of suberin and associated wax in leaf epidermis (Kosma *et al.*, 2014).

The NAC family of transcription factors comprises both transcriptional activators and repressors. A transcription repression function has been specifically demonstrated for calmodulin-binding NAC protein (Kim *et al.*, 2007), the vascular-related NAC-domain-interacting 2 protein (Yamaguchi *et al.*, 2010), and the NAC050 and NAC052 (Ning *et al.*, 2015). Moreover, some NAC-type transcription factors, such as GmNAC20, contain an active repression domain of 35 amino acids (NARD) with a highly conserved sequence among repressor proteins, which includes the hydrophobic LVFY motif also thought to contribute to the repression function (Hao *et al.*, 2010). The alignment of the NARD-like sequence from StNAC103 and proteins showing suppression of transcriptional activity (Hao *et al.*, 2010) shows conservation of the LVFY motif (Supplementary Fig. S8) and 16 identical residues from the 17 highlighted previously by Hao *et al.* (2010) in StNAC103. This is compatible with a repression activity of StNAC103 and is in agreement with the increase of suberin and wax observed in *StNAC103*-silenced lines.

Phellem cells are formed with the activity of a lateral meristem called phellogen, and phellem initials should be elongated and produce thick suberized cell walls before suffering from programmed cell death and becoming mature cork cells, in a similar way to as is described for xylem formation (Hertzberg *et al.*, 2001). *StNAC103* is highly expressed in tissues under the suberization process, but this gene induction is counter-intuitive with the suggested repressor role of this gene, whose silencing yields an increase in suberin and associated wax in *StNAC103-RNAi* periderm. It is probable that the differentiation of phellem cells, as is the case for xylem cells (Schuetz *et al.*, 2012), is a process that is tightly regulated transcriptionally. Hence, it can be surmised that *StNAC103* functions by preventing a premature or inappropriate suberization of phellem initials, probably by competing with other regulatory proteins that promote suberization such as MYB41. The competition of positive and negative stimuli would provide a fine-tuning mechanism to control the suberin deposition process spatially and temporally. There are several cases in the literature in which certain biological processes must be rigorously regulated and the repressor is expressed concurrently with onset of the process. For instance, *EgMYB1*, a key repressor of lignin biosynthesis in *Eucalyptus*, is preferentially expressed during xylem differentiation (Legay *et al.*, 2010). Moreover, in Arabidopsis, the ABA repressor 1 (ABR1), which is a negative regulator of ABA response, is highly responsive to ABA treatment (Pandey *et al.*, 2005), and RAP2.1, a DREB (dehydration-responsive element-binding protein) transcriptional repressor, is induced by drought (Dong and Liu 2010). Apart from its role in phellem cells undergoing suberization, *StNAC103* also turns on in cells involved in wound healing, similarly to the suberin biosynthetic gene *FHT* (Boher *et al.*, 2013). It is widely known that a wound, which is a potential focus of infection due to the loss of the protective barrier, induces biotic and abiotic stress responses in plants. Healing of the wound tuber requires rapid suberization of existing parenchyma cells bordering the wound surface and later development of a deeper wound phellogen layer. The induction of genes involved in suberin deposition, cell wall, and cell proliferation during the first days after wounding in potato tuber has been reported (Neubauer *et al.*, 2012; Lulai and Neubauer, 2014). The induction of *StNAC103* probably mediates the amount of suberin that is synthesized during the formation of this protective healing tissue.

The induction of *StNAC103* in the proliferative cells of lenticular phellogen is consistent with a repression function in cells that should remain undifferentiated without suberin in their cell walls. Lenticels are responsible for gas exchange in the periderm and undergo long-term structural changes by means of the production of non-suberized and suberized filling tissues depending on the physiological and environmental requirements (Hayward, 1974; Groh *et al.*, 2002). The regulation of this gas exchange involves cell proliferation to increase gas exchange and suberin biosynthesis to restrict gas exchange (Lendzian, 2006), and StNAC103 can participate in maintaining this balance. In the same line of evidence, the promoter activity in young roots also reinforces the involvement of StNAC103 in the suggested repressor role. In roots undergoing primary growth, cell wall suberization is confined to the root water barriers: the exodermis and endodermis. Accordingly, the typical suberin genes such as *GPAT5*, *ASFT*, and *FHT* show their promoter activity localized in these suberized cell layers (Beisson *et al.*, 2007; Molina *et al.*, 2009; Boher *et al.*, 2013). In contrast, the *StNAC103* promoter is very active in the apical meristem of potato primary and lateral roots, suggesting a role for this gene in the root meristem, probably preventing a premature suberization in meristematic cells. Remarkably, the lipid transporter ABCG11/WBC11/Desperado, whose putative ortholog is up-regulated in the periderm of *StNAC103-RNAi* lines, is also expressed in the apical and lateral meristems of roots (Bird *et al.*, 2007; Luo *et al.*, 2007; Panikashvili *et al.*, 2007, 2010).

Our results in potato roots showed induction of *StNAC103* by ABA treatment. This is in agreement with the prediction of one ABA-responsive element (ABRE) in the *StNAC103* promoter according to the PLACE and PlantCare databases (Higo *et al.*, 1999; Lescot *et al.*, 2002) but also with the ABA-responsive induction of *StNAC103* in the RNA sequencing (RNA-seq) data from different potato cultivars (Massa *et al.*, 2011; Singh *et al.*, 2013). ABA is known to be involved in suberin deposition. In wound-healing potato tubers, the ABA treatment enhances an increase in the suberin poly(aliphatic) domain (Lulai *et al.*, 2008), a fact that is also supported by the ability of ABA to induce suberin-associated genes such as *GPAT5*, *FHT*, *AtMYB41*, and *ABCG6* (Boher *et al.*, 2013; Kosma *et al.*, 2014; Yadav *et al.*, 2014; Barberon *et al.*, 2016).

Despite the notable increase in the amount of suberin and associated wax, the periderm from silenced *StNAC103* lines did not show any reduction in water permeability. A correlation has been found in several studies between a decrease in suberin or the associated wax load and the impairment of the barrier function in potato tuber periderm and Arabidopsis seeds (i.e. Beisson *et al.*, 2007; Serra *et al.*, 2009*a*). However, the accumulation of suberin monomers has not always been associated with an improvement in the barrier function. For instance, ectopic accumulation of suberin monomers in the leaf cuticle has been reported as even resulting in an impairment of the water barrier function (Li *et al.*, 2007; Cominelli *et al.*, 2008; Kosma *et al.*, 2014). These mixed findings have led to the suggestion that in addition to the monomer load, the structural organization may also be crucial for the water permeability of the apoplastic barrier.

We do not know the direct target genes of StNAC103, but four genes involved in several key steps of wax and suberin accumulation (*KAR*, *FHT*, *CYP86A33*, and *WBC11*) have been transcriptionally up-regulated in the periderm of *StNAC103-RNAi* lines. The induction of these genes is in agreement with the modified lipid profile observed in the periderm of *StNAC103*-silenced lines. For example, *KAR* is involved in the *de novo* synthesis of fatty acids (Li-Beisson *et al.*, 2013) and its activation meets the need of a higher demand for fatty acids. Taking into consideration the phenotype of the mutants (Gou *et al.*, 2009; Molina *et al.*, 2009; Serra *et al.*, 2010), the up-regulation of *FHT* supports the increase in wax ferulate esters, ω-hydroxyacids, primary alcohols, and ferulic acid observed. The up-regulation of *CYP86A33*, based on the chemical composition of *CYP86A1* and *CYP86A33* mutants (Li *et al.*, 2007; Höfer *et al.*, 2008; Serra *et al.*, 2009*b*), is in agreement with the increased presence of the two most abundant suberin monomers, C18:1 ω-hydroxyacid and diacid. The induction of ABCG11/WBC11, a key component of the export pathway for cutin, wax, and suberin, agrees with the accumulation of alkanes, primary alcohols, and C18:1 ω-hydroxyacid suberin monomers (Bird *et al.*, 2007; Luo *et al.*, 2007; Panikashvili *et al.*, 2007, 2010; Ukitsu *et al.*, 2007).

In conclusion we report *StNAC103* as a candidate gene for suberin and associated wax repression that probably acts by means of transcriptional regulation of genes related to suberin and wax accumulation. *StNAC103* is induced not only in cells undergoing suberization, but also in cells where suberization does not take place but which are physically close to suberized cells, such as lenticular phellogen and root apical meristem. Taking into account the findings presented, it is tempting to speculate that the activation of *StNAC103* has a common goal: to prevent suberin deposition. This control can occur either in cells where premature deposition of suberin can be detrimental to the proper functioning of the tissue or as a suberin deposition brake in cells where suberin accumulation takes place, suggesting that the deposition of apoplastic lipids must be under tight control. Further research is needed to elucidate the direct targets of StNAC103 transcription factor. To the best of our knowledge, this is the first regulatory gene characterized as having a role in the repression of suberin and associated wax.

## Supplementary data

Supplementary data are available at *JXB* online.

Figure S1. StNAC103 protein and nucleotide sequences showing the NAC and TAR domains and the region used for silencing.

Figure S2. *StNAC103* and *FHT* promoter activity in potato primary root.

Figure S3. Relative transcript abundance of *StNAC057* in *StNAC103-RNAi* and wild-type periderm.

Figure S4. Wax fatty acids, primary alcohols, and feruloyl esters of primary alcohols in *StNAC103-RNAi* and the wild type.

Figure S5. Wax composition plotted for the different lines of *StNAC103-RNAi* analyzed.

Figure S6. Suberin composition for the different lines of *StNAC103-RNAi* analyzed.

Figure S7. Water permeance of *StNAC103*-silenced periderm from tubers stored for 39 d.

Figure S8. Alignment of the NARD-like sequences from StNAC103 (99–134) and proteins showing suppression of transcriptional activity.

Table S1. List of genes, loci, and primers used.

## Supplementary Material

Supplementary Table

Supplementary Figures

## Abbreviations:

ABA: abscisic acid
ABCG11/WBC11: ATP-binding cassette/white–brown complex
AtMYB41: myeloblastosis transcription factor
CYP86A33: cytochrome P450
FHT: fatty ω-hydroxyacid/fatty alcohol hydroxycinnamoyl transferase
KAR: ketoacyl-ACP reductase
NAC: NAM, ATAF, and CUC
NARD: NAC repression domain
qPCR: quantitative PCR
RT–PCR: reverse transcription–PCR.
