# Supplementary material for: Silencing of the potato StNAC103 gene enhances the accumulation of suberin polyester and associated wax in tuber skin
Source: J Exp Bot. 2016 Aug 12;67(18):5415–27. doi: 10.1093/jxb/erw305 (PMC5049391; doi:10.1093/jxb/erw305)
Supplement: Supplementary Figures [file erw305_suppl_supplementary_figures_s1_s8.pdf]

Supplementary information (SI) appendix

Silencing of the potato tuber skin-induced *StNAC103* gene enhances accumulation of suberin and suberin-associated wax

Roger Verdaguer[ǂ], Marçal Soler[ǂ], Olga Serra, Aïda Garrote, Sandra Fernández, Dolors Company-Arumí, Enriqueta Anticó, Marisa Molinas, Mercè Figueras[*].

[ǂ] these authors contributed equally to this work

[*]Author for correspondence: merce.figueras@udg.edu

Supplementary Imformation (SI) contains:

SI Figures 8

SI Table 1

A)

```
StNAC103                     MEENLPPGFRFHPTDEELITYYLNNKVSDFNFTARAITDVDLNKSEPWDL 50
StNAC103 Group Phureja       MEENLPPGFRFHPTDEELITYYLNNKVSDFNFTARAITDVDLNKSEPWDL 50
                             **************************************************

StNAC103                     PAKASMGEKEWYFFSLKDRKYPTGLRTNRATEAGYWKTTGKDKEIFRGGT 100
StNAC103 Group Phureja       PAKASMGEKEWYFFSLKDRKYPTGLRTNRATEAGYWKTTGKDKEIFRGGT 100
                             **************************************************

StNAC103                     GVLVGMKKTLVFYRGRAPKGEKTNWVMHEYRIETRFGYKPSKEEWVVCRV 150
StNAC103 Group Phureja       GVLVGMKKTLVFYRGRAPKGEKTNWVMHEYRIETRFGYKPSKEEWVVCRV 150
                             **************************************************

StNAC103                     FQKSSTVKKPQPTSSSPLSLESPCDTNYTIANELGDIELPFNFNYLTTTP 200
StNAC103 Group Phureja       FQKSSTVKKPQPSSSSPLSLESPCDTNYTIANELGDIELPFNFNYLTTTP 200
                             ************:*************************************

StNAC103                     SSTINNISLHNYNNDNINLAAAAAA-TREAAAANTHPLLPWSSNLVSSNL 249
StNAC103 Group Phureja       SSAINNISLHNYNNDNINLAAAAAAATREAAAANTHPLLPWSSNLLSSNL 250
                             **:********************** *******************:****

StNAC103                     SSVNSLLFRALQLRGYSPREQATSSTTTTHDYAFMLPQENILTTQFGNDF 299
StNAC103 Group Phureja       SSVNSLLFRALQLRGYSPREQATSSTTTTHDYAFMLPQENILTTQFGNDF 300
                             **************************************************

StNAC103                     AVNSIGAPSSSMALDNSVQHQQPQEQLYKLDSDIL- 334
StNAC103 Group Phureja       AVNSTGAPSSSMALDNSVQNQQPQEQLYKLDSNIW- 335
                             **** **************:************:*
```

B)

```
StNAC103         1     ATGGAGGAAAATCTTCCTCCAGGGTTCAGG   30
Group Phureja    31    TTCCATCCAACAGATGAAGAACTCATTACT   60
                 61    TATTATCTGAATAATAAAGTTTCTGATTTC   90
                 91    AACTTCACTGCTAGAGCTATTACTGATGTT   120
                 121   GATCTCAATAAGTCTGAGCCTTGGGACCTC   150
                 151   CCTGCAAAAGCGTCAATGGGAGAAAAAGAA   180
                 181   TGGTATTTCTTCAGTCTAAAAGATCGAAAG   210
                 211   TATCCAACAGGACTTCGAACAAACAGAGCT   240
                 241   ACAGAAGCAGGCTACTGGAAAACAACAGGG   270
                 271   AAGGATAAAGAGATATTTCGTGGTGGAACG   300
                 301   GGAGTCCTTGTTGGGATGAAGAAAACCCTA   330
                 331   GTTTTCTACAGAGGAAGAGCTCCTAAGGGT   360
                 361   GAAAAAACCAATTGGGTTATGCATGAATAC   390
                 391   AGAATTGAAACAAGATTTGGTTACAAACCT   420
                 421   TCTAAGGAGGAATGGGTAGTCTGCAGGGTG   450
                 451   TTCCAAAAGAGTTCAACTGTGAAAAAGCCA   480
                 481   CAACCAACATCATCTTCTCCTCTATCCCTA   510
                 511   GAGTCACCTTGTGACACTAATTACACAATA   540
                 541   GCAAATGAGCTTGGAGATATTGAGCTACCA   570
                 571   TTTAATTTCAACTACCTAACCACTACTCCA   600
                 601   TCAAGCACGATCAACAATATTTCCTTGCAT   630
                 631   AATTACAACAACGATAATATAAACTTGGGC   660
                 661   TGCTGCTGCTGCTGCAACAAGAGAAGCAGC   690
                 691   AGCAGCAAACACTCATCCATTACTACCTTG   720
                 721   GTCTTCGAACTTGGTAAGCTCTAATCTTTC   750
                 751   ATCAGTAAATTCATTGCTTTTTAGGGCATT   780
                 781   ACAATTAAGGGGTTATTCGCCAAGAGAACA   810
                 811   AGCTACAAGTTCTACTACTACTACGCATGA   840
                 841   CTACGCGTTTATGCTCCCACAGGAGAATAT   870
                 871   TCTCACAACGCAGTTTGGAAATGATTTTGC   900
                 901   TGTGAATAGTATTGGGGCACCTTCTTCATC   930
                 931   TATGGCATTAGATAATTCTGTACAGCATCA   960
                 961   ACAGCCACAGGAACAATTGTaCAAAtTGGA   990
                 991   CTCCGATATTTTGTGA                 1006
```

Supplementary Fig. 1. (A) Amino acid sequence alignment between the sequence obtained of *StNAC103* from *S. tuberosum* Group Tuberosum (KT598221) and from *S. tuberosum* Group Phureja (PGSC0003DMP400009522). The NAC domain is at N-terminal end and is shown shadowed in grey. The TAR region is the remaining protein sequence at the C-terminal end. **(B)** *StNAC103* Group Tuberosum nucleotide sequence showing the sequence used to generate the *StNAC103-RNAi* hairpins is highlighted in orange characters. NAC domain is also shadowed in grey.

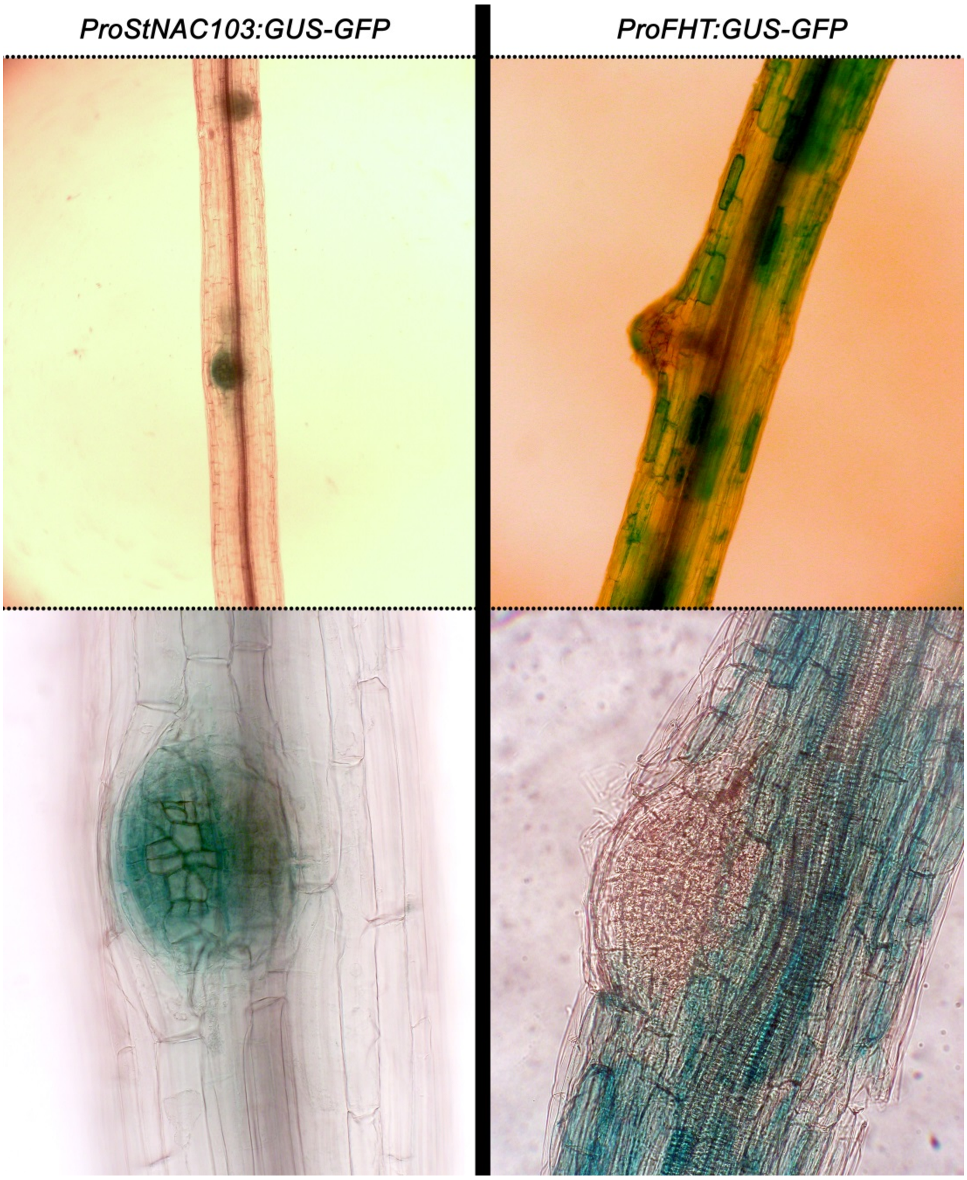

Supplementary Fig. 2. *StNAC103* and *FHT* promoter activity in potato primary root. Plants were grown in hydroponics and roots were GUS stained in parallel.

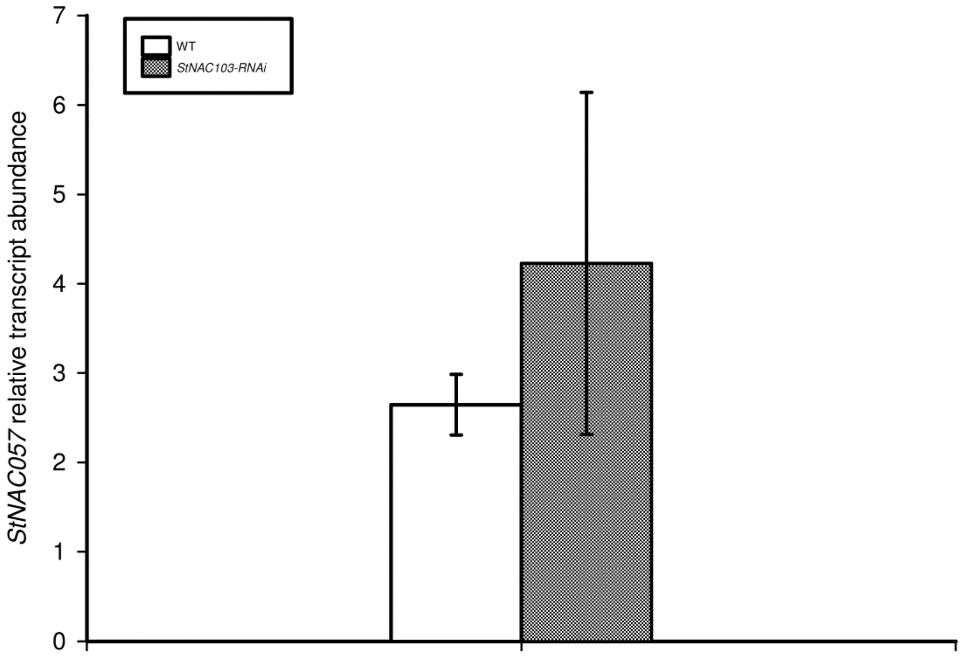

Supplementary Fig. 3. The relative transcript abundance of *StNAC057* in *StNAC103-RNAi* and wild-type periderms was analyzed by microfluidics qPCR. Values are the mean ± SD of wild-type (four biological replicates) and two independent transformation events for *StNAC103-RNAi*, lines 43 and 45 (five and four biological replicates, respectively). *The StNAC057* transcript accumulation did not show statistically significant differences between *StNAC103-RNAi* and wild-type periderms (t test; p=0.14).

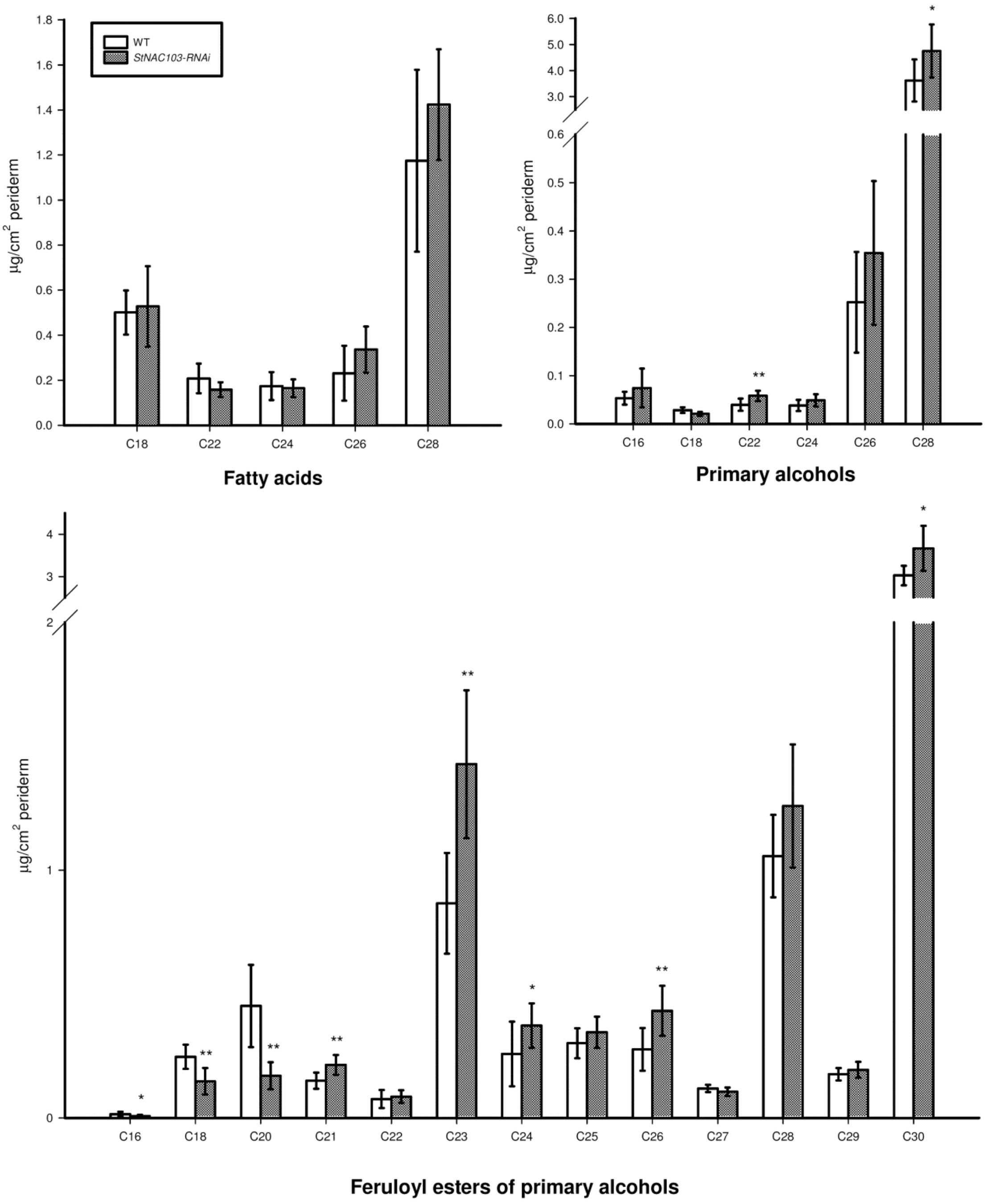

Supplementary Fig. 4. Chemical composition of wax fatty acids, primary alcohols and feruloyl esters of primary alcohols in *StNAC103-RNAi* and wild-type periderms. Values are the mean ± SD of wild-type (n=5) and two independent transformation events for *StNAC103-RNAi* (n=4, n=3). Statistical significant differences (p<0.05) are denoted with one asterisk whereas pronounced statistical significant differences (p<0.01) are shown with two asterisks.

StNAC103-RNAi lines

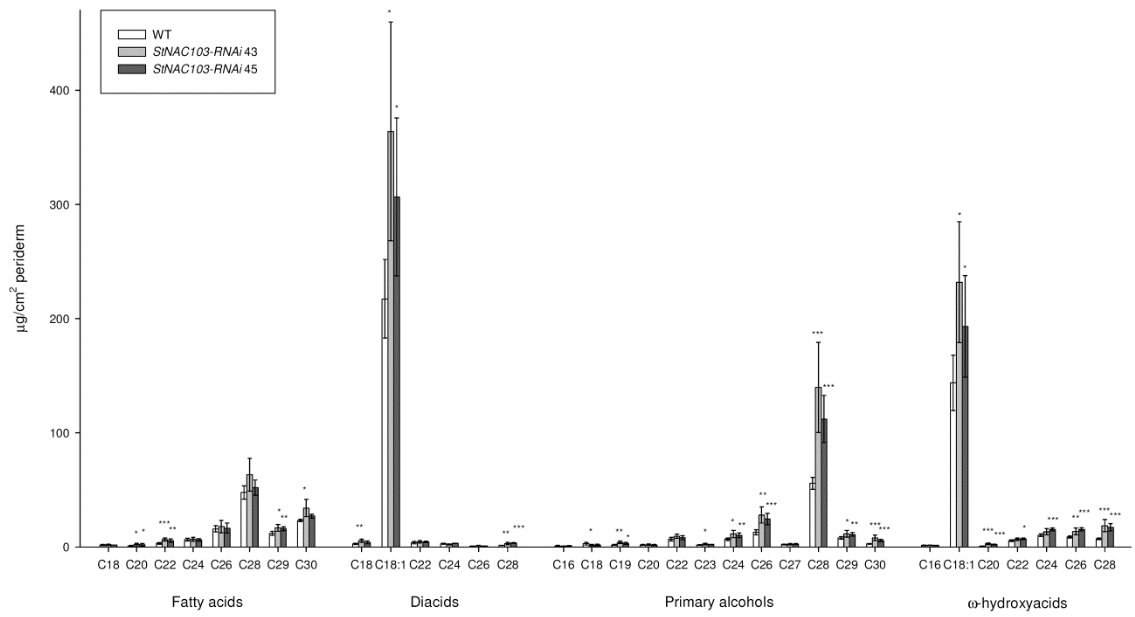

Supplementary Fig. 5. Wax chemical composition plotted for the different lines of *StNAC103-RNAi* and wild-type analyzed. Values are the mean ± SD of wild-type (n=5), *StNAC103-RNAi 43* (n=4), *StNAC103-RNAi 45* (n=3). Statistical significant differences ($p<0.05$) are denoted with one asterisk whereas pronounced statistical significant differences ($p<0.01$ and $p<0.001$) are shown with two and three asterisks respectively.

StNAC103-RNAi lines

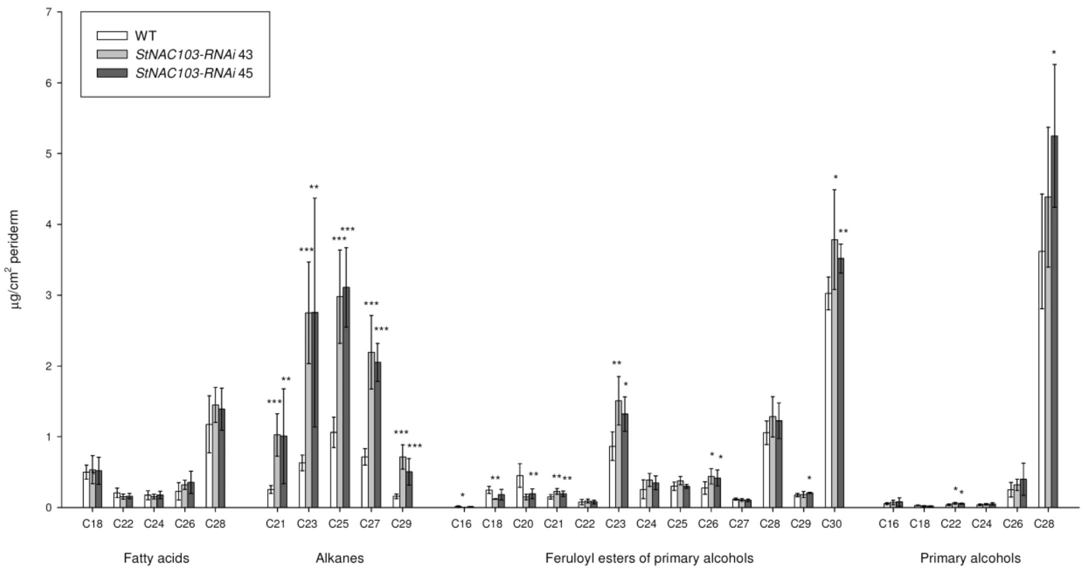

Supplementary Fig. 6. Suberin chemical composition for the different lines of *StNAC103-RNAi* and wild-type analyzed. Values are the mean ± SD of wild type (n=5), *StNAC103-RNAi 43* (n=4), *StNAC103-RNAi 45* (n=3). Statistical significant differences ($p<0.05$) are denoted with one asterisk whereas pronounced statistical significant differences ($p<0.01$ and $p<0.001$) are shown with two and three asterisks respectively.

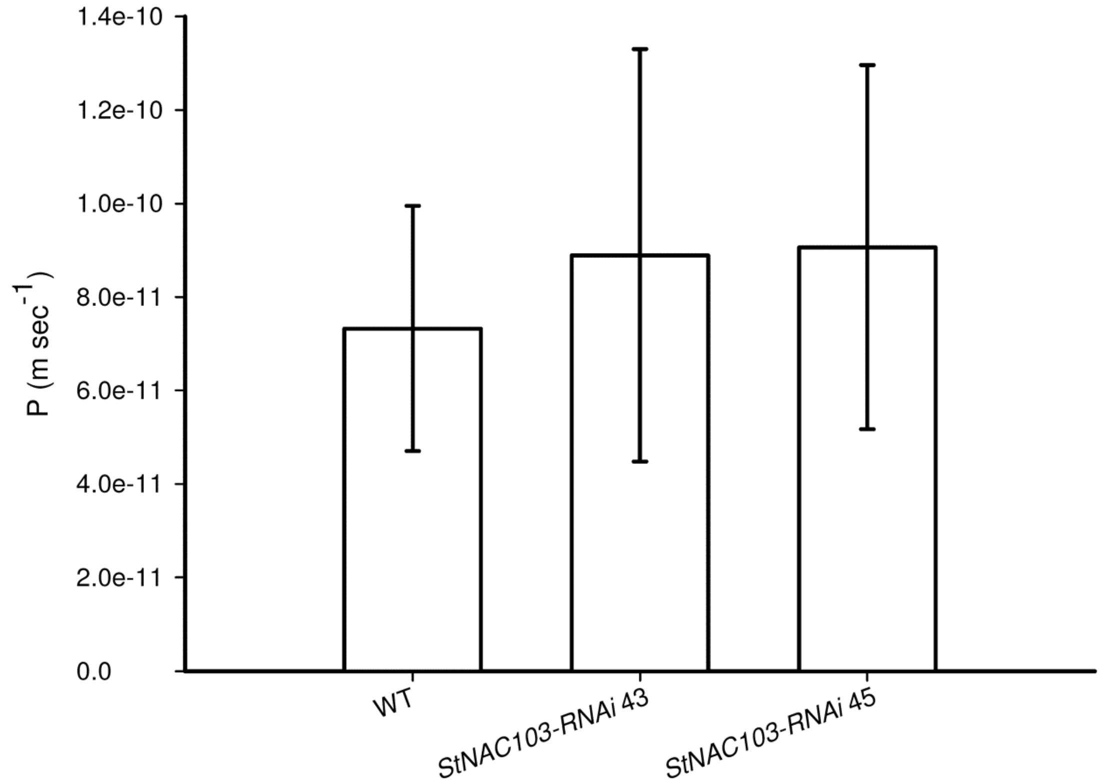

Supplementary Fig. 7. Periderm water permeance in *StNAC103-RNAi* and wild-type lines. Periderms were isolated from 39-d-stored tubers. Values are the mean ± SD of 3-7 biological replicates.

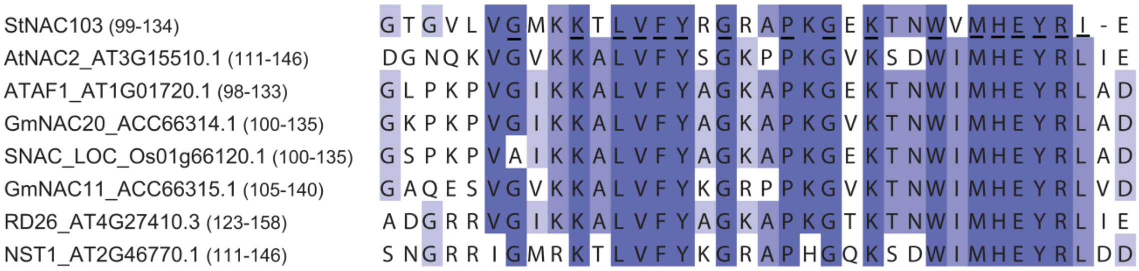

Supplementary Fig. 8. Alignment of the NARD-like sequences from StNAC103 (99-134), GmNAC35_ACC66316.1 (101-136), AtNAC2_AT3G15510.1 (111-146), ATAF1_AT1G01720.1 (98-133), GmNAC20_ACC66314.1 (100-135), SNAC_LOC_Os01g66120.1 (100-135), GmNAC11_ACC66315.1 (105-140), RD26_AT4G27410.3 (123-158), NST1_AT2G46770.1 (111-146) proteins. Identical residues identified by Hao *et al.*, (2010) were underlined.

Hao Y-J, Song Q-X, Chen H-W, Zou H-F, Wei W, Kang X-S, Ma B, Zhang W-K, Zhang J-S, Chen S-Y. 2010. Plant NAC-type transcription factor proteins contain a NARD domain for repression of transcriptional activation. Planta **232**, 1033–1043.
